# The Story of the Insane from Year to Year

**Published:** 1891-01-17

**Authors:** 


### THE STORY OF THE INSANE FROM YEAR TO YEAR.

### EAST RIDING ASYLUM, BEVERLEY.

On the last day of the year 1889 there were resident 271 patients, being a decrease of 20 on the numbers for the previous year, and this decrease was accounted for by the transfer of patients to other asylums; by a decreased admission rate, and by an increased death-rate—the former being 51 in 1889, as against 69 in 1888, and the latter 34, as against 22. The recoveries stand at 31·25 per cent. on the admissions. Of the 19 men admitted, we are told that two were congenitally affected, 11 had organic brain disease, two were over 60 years of age, two had been insane for over a year, and only two could be considered curable. The asylum was visited by enteric fever —six patients being stricken by the disease, and all recovered from it. The visitors record their favourable opinion of Dr. Macleod's services. The weekly rate is 8s. 6d.

### ROYAL ASYLUM, PERTH.

This is one of the Scotch institutions of which we have more than once said that England would be better if it possessed similar ones. The Perth Asylum is now 63 years old, or, at least, it has just issued its sixty-third annual report. From this report we learn that the total number under treatment during the year was 129, and that the average daily number resident was 98; the number at the close of the year being exactly 100. Nine of these are voluntary boarders. The percentage of recoveries is high, namely, 41·66 on the admissions; and the death-rate is low—7·13 on the average number resident. We think the following extract from Dr. Urquhart's report is worthy of wide circulation: "It is now fully recognized that science and art must be laid under contribution to brighten, to interest, to regulate, and to cure. We seek to strike chords of mind responding to the sense of colour, proportion, right—to harmonize faculties jangled out of tune. Therefore it becomes necessary to devote so much valuable time to the problems of asylum architecture, the occupations and the amusements of the patients, as well as to questions of purely medical treatment and microscopic pathology." The weekly cost of maintenance was £1 19s. 1d. per head; the average rate of payments made by patients is £82 14s. per annum. Some patients, of course, pay much higher sums, and the ordinary minimum rate of board is £60 per annum; but not fewer than 39 patients were maintained at rates varying from £30 to £52, a convincing proof of the boon this institution must be to many of the middle classes.

### SUFFOLK COUNTY ASYLUM.

During the year 1889, 132 patients were admitted, 67 were discharged, and 63 died. There remained on the last day of December, 479 patients. The recoveries reached the creditable percentage of 46·15, but the death-rate was above the average, being 13 per cent. on the average number resident. Nineteen of the deaths were caused by phthisis, 6 by dysenteric diarrhœa, and 3 to enteric fever. Dr. Eager's report is a long and exhaustive one. When speaking of the proposal of the London County Council to establish a hospital for the treatment of insanity, he says: "I am quite at a loss to know why it was expected that a higher recovery rate would result from having a number of acute cases collected together in a hospital, attended by consulting physicians and surgeons ignorant of the treatment and management of mental cases. The Committee appointed to investigate this question considered that the proposed hospital was so eminently desirable that they studiously avoided examining asylum superintendents and other witnesses whose testimony was known to be unfavourable to the proposed hospital. They, moreover, decided that in insanity they would accept the existence of brain disease only, and that successful results would follow a treatment of drugs or operative interference. The idea of moral treatment, good nursing, and hygiene providing a means towards recovery, they appeared to totally ignore."

